# Genetic fingerprinting of salmon louse (*Lepeophtheirus salmonis*) populations in the North-East Atlantic using a random forest classification approach

**DOI:** 10.1038/s41598-018-19323-z

**Published:** 2018-01-19

**Authors:** A. Jacobs, M. De Noia, K. Praebel, Ø. Kanstad-Hanssen, M. Paterno, D. Jackson, P. McGinnity, A. Sturm, K. R. Elmer, M. S. Llewellyn

**Affiliations:** 1 Institute of Biodiversity, Animal Health & Comparative Medicine, College of Medical, Veterinary & Life Sciences, University of Glasgow, Glasgow, UK; 2 Norwegian College of Fishery Science, UiT The Arctic University of Norway, N-9037 Tromsø, Norway; 3 Ferskvannsbiologen Ltd, Lødingen, Norway; 4 Department of Biology, University of Padova, Padova, Italy; 5 Marine Institute, Galway, Ireland; 6 School of Biological, Earth and Environmental Sciences, University College Cork, Cork, Ireland; 7 Institute of Aquaculture, University of Stirling, Stirling, UK

## Abstract

Caligid sea lice represent a significant threat to salmonid aquaculture worldwide. Population genetic analyses have consistently shown minimal population genetic structure in North Atlantic *Lepeophtheirus salmonis*, frustrating efforts to track louse populations and improve targeted control measures. The aim of this study was to test the power of reduced representation library sequencing (IIb-RAD sequencing) coupled with random forest machine learning algorithms to define markers for fine-scale discrimination of louse populations. We identified 1286 robustly supported SNPs among four *L. salmonis* populations from Ireland, Scotland and Northern Norway. Only weak global structure was observed based on the full SNP dataset. The application of a random forest machine-learning algorithm identified 98 discriminatory SNPs that dramatically improved population assignment, increased global genetic structure and resulted in significant genetic population differentiation. A large proportion of SNPs found to be under directional selection were also identified to be highly discriminatory. Our data suggest that it is possible to discriminate between nearby *L. salmonis* populations given suitable marker selection approaches, and that such differences might have an adaptive basis. We discuss these data in light of sea lice adaption to anthropogenic and environmental pressures as well as novel approaches to track and predict sea louse dispersal.

## Introduction

Caligid sea lice are copepod ectoparasites of marine fish. In the northern hemisphere, the salmon louse (*Lepeophtheirus salmonis*) is the species most commonly infecting farmed and wild salmonids^1^, at considerable cost to animal health, biodiversity security, and economic growth. Conservative estimates of costs and losses attributed to sea louse infections, (estimated at €350 M million in 2014 in Norway alone^2^ suggest these are the single greatest pathogen burden on the global salmonid aquaculture industry. The life cycle of the sea louse involves high levels of replication, dispersal and obligate host-association^1^; this means that local environmental conditions, sea currents, and population densities are important ecological and demographic conditions to facilitate or impede infestation^1,3,4^. Eggs carried by females hatch to free-swimming non-feeding nauplii, planktonic larvae that are passively dispersed. These nauplii undergo two moults before developing into a free swimming copepodid. Development time is temperature dependent and at 10 °C the infectious copepodid stage, which needs to settle successfully on a host for survival, develops two to three days post hatching. During the host-associated phase of the lifecycle, which progresses through further larval and preadult stages before reaching the reproducing adult stage, salmon lice feed on mucus, skin and blood of their host fish^1^. Depending on severity, infections can cause skin lesions, anaemia, osmoregulatory dysfunction, stress, suppression of growth and immune function, secondary infections and, if left untreated, mortality^1,5^. Salmon louse control has traditionally relied on a limited number of drug treatments^5,6^, but large-scale reliance on just a few products is associated with a significant risk of developing drug resistance^5,7^.

Understanding and predicting salmon louse dispersal is a crucial element for predicting infestation, connectivity and the spread of salmon lice and associated drug resistance alleles. There have been several attempts to characterize population genetic structure in *L. salmonis* in the North Atlantic using conventional microsatellite and sequence markers^8–12^. High gene flow between sites is consistently reported. In the largest such study (13 microsatellite loci, 2500 samples), significant but weak (0.0022) F_ST_ was detected across the Atlantic, with no evidence for population genetic structuring within geographic regions. More recently a genome-wide SNP array was developed and deployed using 5091 variable markers, and showed similar results in terms of population structure, alongside extensive evidence of selective sweeps and linkage disequilibrium attributable, at least in part, by the use of chemotherapeutics in aquaculture^13^. Besnier *et al*., 2014 also included a linkage map for these markers onto which the current assemble of the louse genome has been superimposed (https://metazoa.ensembl.org/Lepeophtheirus_salmonis/Info/Index). Thus, although significant progress has been made in determining population genetic signatures of selection in *L. salmonis*, the goal of distinguishing louse populations occurring in different regions - a valuable component of detecting dispersal of lice between farms – remains expensive and difficult.

Determining genetic structure in pelagic marine species has always been challenging. High rates of adult and larval dispersal impede the accumulation of neutral variation among populations and regions. Nonetheless, several studies have achieved genetic stock delineation by focusing on non-neutral or putative adaptive markers in conjunction with high numbers of SNP markers (e.g^14,15^.). In extreme cases like *Anguilla rostrata*, where the organism’s reproductive ecology predicts and the genetic data support panmixia among different populations, the challenge of determining genetic differences between different populations is even greater^16,17^. Screening thousands of variable SNP markers against population genetic summary statistics may be able to detect outliers, however the identification of which markers might best assign individuals to their appropriate populations, groups, or ecomorphs necessitates further computational approaches. To this end, population genetics can usefully borrow from machine learning algorithms developed in the context of genome-wide association studies^18^. Such approaches have been successfully used in *A. rostrata*, to identify SNPs that predict rearing habitat as the result of intra-generational selection^19^, for example. More recently similar approaches have been employed to successfully discriminate *Salmo salar* populations^20^.

In this study, we identify population structure and loci under selection in *L. salmonis* using high throughput SNP genotyping and advanced analytical methods. To achieve this we collected *L. salmonis* from four different sites in the North-Eastern Atlantic (UK, Norway and two sites in Ireland) and generated genomic SNP data using a IIB restriction-enzyme associated library preparation approach^21^. We then tested the power of Random Forest machine learning to reveal population structure and find the method reveals previously un-recognized population differences and fine-scale population differentiation.

## Methods

### Sample collection and DNA isolation

**Figure 1:**
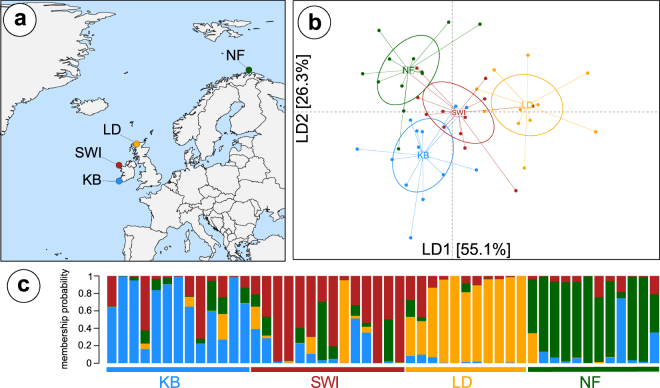
Population structuring in *L. salmonis* bases on the full SNP dataset. (**A**) Map showing the sampling sites of all four populations across the North-East Atlantic: Finnkirka (NF), Loch Duart (LD), Kenmare Bay (SWI) and Kilkieran Bay (KB). (**B**) DAPC plot of the first and second linear discriminant axis based on the full SNP dataset, explaining a total of 81.4% of the total variation. (**C**) Membership probability plot showing the population assignment probability for each individual. Shapefiles (for maps, rivers and lakes) were downloaded from natural earth (http://www.naturalearthdata.com/downloads/) and plotted in R. All data and software are open source.

Adult and pre-adult *Lepeophtheirus salmonis* were collected in four sites around the North-East Atlantic from 18–24 month old Atlantic Salmon from commercial pens in 2015. Sites included Finnkirka (NF), Lebesby, Norway; Loch Duart (LD), Scotland, UK; Kenmare Bay (SWI), Kenmare, Ireland and Kilkieran Bay (KB), Galway, Ireland (Fig. 1A). Male and immature female individuals only were selected for sequencing to avoid gamete contamination of DNA extracts. DNA was obtained using a modified salt extraction protocol, quantified using a NanoDrop® ND-1000 spectrophotometer and visualised on a 1.5% agarose gel to assess quality. Fifteen high quality (260/280 ratio ~1.8) and high molecular weight extracts were chosen per site.

### IIb-RAD library preparation and sequencing

Library preparation was undertaken as described in Wang *et al*. 2012^21^. By reference to *in silico* digestion of the *L. salmonis* reference genome (genome available at https://metazoa.ensembl.org/Lepeophtheirus_salmonis/Info/Index) two enzymes were selected based on potential coverage: AlfI (restrictions site ^5′(10/12)GCA(N)_6_ TGC(12/10)3′^) and CspCI (restriction site ^5′(11/12)CAANNNNNGTGG(12/13)3′^). Digested DNA of each sample was ligated to a pair of partially double-stranded adaptors with compatible and fully degenerated overhangs (5′NNN3′). Finally, the obtained IIb-RAD tags were amplified to introduce a sample-specific 7 bp barcode and the Illumina NGS annealing sites using two different pairs of sequencing primers. A 1.8% agarose gel electrophoresis of the PCR products was performed to verify the presence of the expected 150 bp target band (fragment, barcodes and adaptors included). In order to ensure an approximately equimolar contribution of each sample to the library, the concentration of each PCR product was measured from the intensity of the target band in a digital image of the 1.8% agarose gel. We prepared two libraries in total, one for each IIB-REase. The purification of the libraries from high-molecular weight fragments and primer-dimers was achieved first by removing the target band on agarose gel library and eluting them in water overnight; followed by DNA capture with magnetic beads (SPRIselect® Beckman Coulter). The DNA concentration in the purified libraries was quantified with a Qubit® Fluorometer (Invitrogen) and the libraries were combined in one single pool. Two library pools were sequenced, first on a NextSeq 500, then on a MiSeq (Illumina, San Diego, CA, USA) with a single 1 × 50 bp setup using ‘Version2’ chemistry at Glasgow Polyomics (www.polyomics.gla.ac.uk), which also implemented the read demultiplexing and quality-filtering.

### IIb-RAD data processing

Short reads were aligned to the reference genome in bowtie 2^22^ and SNPs were called using the STACKS v1.42 package with a minimum read depth of 3^23^. The *rxstacks* module was used to further screen SNPs and the *population* module filter and export genotypes with a minimum depth of coverage of 6, minimum minor allele frequency of 0.05, maximum observed heterozygosity of 0.5 and present in at least 60% of individuals. To avoid sequence artefacts generated by low complexity in restriction enzyme recognition site, SNPs at positions 12–26 were excluded from the analysis. For those RAD tags that retained diversity after screening for artefacts, only a single SNP per locus was selected for subsequent analysis.

### Population structure analysis and detection of positive selection

Principal components analysis (PCA), discriminant analysis of principal components (DAPC), and population assignment probabilities were calculated in *adegenet*^24^. Analyses of molecular variance (AMOVA), Weir and Cockerham estimators of FST, and summary statistics (Ho, He, Gis, π) were calculated in *Genodive*^25^. P-values for F_ST_ were FDR adjusted for multiple comparisons using a Benjamini-Hochberg correction in the R-package *p-adjust*. Isolation-by-distance was assessed using a Mantel test implemented in the *vegan* R-package. Loci putatively under positive selection were identified in Lositan^26^ using a FDR < 0.1 significance threshold and localised on the *L. salmonis* linkage map (Glover, K *Pers Comm*) to assess genomic correspondence with a previous population genomic study^13^. Lositan results were plotted using in R. Further, we performed a second outlier analysis using BayeScan, as it has a lower type I error rate compared to Lositan^27^). We ran BayeScan with prior odds of 100 due to the small number of SNPs and detected significant outliers with a FDR threshold of 0.05 and putative outliers with a FDR threshold of 0.1. Finally, we post hoc identified overlapping outlier SNPs between BayeScan and Lositan.

In order to identify genes potentially under positive selection we identified all genes within a 10 kb region around each outlier SNP by blasting the sequence against the *L. salmonis* reference genome using the *blastn* function in the EnsemblMetazoa database. We identified all genes within those 10 kb regions and when possible determined their function using the *UniprotKB* database.

### Using Random Forest analysis to detect population-discriminatory SNPs

In order to detect SNPs characteristic of each population we employed a tree-based ensemble machine learning approach using the *randomForest* package in R. Populations were numerically coded and missing data imputed using the *na.roughfix* command. Three independent random forest runs with 100,000 trees each were conducted and checked for convergence between runs by performing Pearson correlation between SNP importance values. The random forest algorithm randomly subdivides the full dataset into a training dataset (66.6%), which is used to train the algorithm, and a test dataset (‘Out-of-bag’; 33.3%) that is used to test the classification success of a tree. For each tree, the dataset gets subdivided into new subsets and the number of trees for which the out-of-bag (OOB) error rate stabilises was chosen, reducing the risk of overfitting. The resultant ranked dataset (R^2^ > 0.95) was used to select a final dataset for the backwards purging approach. As in Laporte *et al*., all loci with an importance < 0 were removed as non-discriminatory^19^. Backwards purging was performed on the remaining 317 SNPs. As such each random forest run was re-implemented (three independent iterations) and after each run the SNP with the lowest importance was removed until only two SNPs were left. We determined the subset of SNPs with the highest discriminatory power based on the lowest OOB error rate, meaning the highest rank reliability of important markers, and we used this subset for further downstream analysis^18,19^.

In order to assess the population discriminatory power of the random forest selected SNPs we used the same methods as for the full SNP dataset. First, we performed a PCA and DAPC in *adegenet* in *R* to visualise population structuring and assess the population assignment accuracy. Second, we performed an AMOVA and estimated pairwise Weir and Cockerham’s Fst in *Genodive*. We also identified the overlap between highly discriminatory SNPs and SNPs potentially under positive selection to assess the impact of selection on discriminating *L. salmonis* populations.

### Validating the use of random forest for population discrimination in larger datasets

In order to determine if a random forest approach also refines population assignment on larger datasets with more individuals and loci, we further tested this algorithm on the SNP dataset published by Besnier *et al*. (2014)^13^. Besnier *et al*. (2014)^13^ genotyped 547 salmon lice from 12 sampling sites from 6 geographic regions for 5091 SNPs using a custom SNP array. Missing data were imputed using the na.roughfix command. We assessed the population assignment success using the DAPC approach for the full dataset before using the random forest algorithm to identify the subset of SNPs with the highest discriminatory power between sampling regions (Canada, Faroe, Shetland, Ireland, Southern and Northern Norway). We ran three independent random forests with 10,000 trees each and ranked SNPs based on their average importance, measured by the ‘Mean Decrease Accuracy’ (MDA). Due to the larger number of SNPs, we selected all SNPs above the lower end of the elbow in the importance value distribution (MDA of 1.5; Figure S3) for the backwards purging step. We determined the subset of SNPs with the highest discriminatory power as the subset of SNPs that minimised the OOB error rate. Similar to the complete dataset, we determined the assignment success of this dataset using the DAPC approach implemented in *adegenet*.

## Results

### Bioinformatic processing & summary statistics

Using IIb-RAD sequencing we generated an average of 1,496,567 ± 673,594 reads per individual for 50 individuals from four populations across the North-East Atlantic (Fig. 1a). The final catalogue contained 111,090 RAD tags with an average coverage of 19.6 ± 6.9 per individual, covering 0.34% of the genome. After stringent filtering we retained 1286 SNPs, spanning 787 different reference genome contigs. Genetic diversity, measured as nucleotide diversity (π) and observed heterozygosity (Ho) were similar across populations (Table 1). Tajima’s D did not indicate any signals characteristic of significant population expansion (Figure S1, Supplementary data).

**Table 1:** Summary table.

Population	N	Mean Coverage	Ho	He	Gis	π
KB	13	19.7	0.278	0.304	0.086	0.3025986
SWI	14	18.6	0.265	0.304	0.128	0.3019599
LD	11	19.8	0.258	0.298	0.132	0.2952346
NF	12	20.2	0.267	0.312	0.143	0.3096919

Summary of sample sizes, mean sequencing coverage per individual and summary statistics, namely observed heterozygosity (Ho), expected heterozygosity (He), inbreeding coefficient (Gis) and genetic diversity (π).

### Population structure using the full SNP dataset

In a first approach, we assessed population genetic structure using the full dataset of 1286 SNPs by several different approaches. A PCA did not reveal any population structuring across the entire range, however using pre-defined populations in the DAPC approach revealed a weak population structuring (Fig. 1b & c, Figure S2, Supplementary data). The population assignment probability was on average 0.82 ± 0.10. An AMOVA showed weak but significant population structure (F_sc_ = 0.018, p < 0.0001; Table 2). However, based on pairwise F_st_ values only LD and KB were significantly genetically differentiated (F_st_ = 0.01, p < 0.0001). No significant isolation-by-distance was detected (R^2^ = −0.35, P = 0.67).

**Table 2:** AMOVA results showing the global population structure.

	Source of Var.	Nested in	% Var	F-stat	F-value	P-value
Full SNP dataset	Within Ind.	—	88.4	F_it	0.116	—
	Among Ind.	Population	9.8	F_is	0.1	p < 0.0001
	Among Pop.	—	1.8	F_sc	0.018	p < 0.0001
Discriminatory SNPs	Within Ind.	—	78.9	F_it	0.211	—
	Among Ind.	Population	11.3	F_is	0.125	p < 0.0001
	Among Pop.	—	9.8	F_sc	0.098	p < 0.0001

### Using machine learning to define population genetic structure

**Figure 2:**
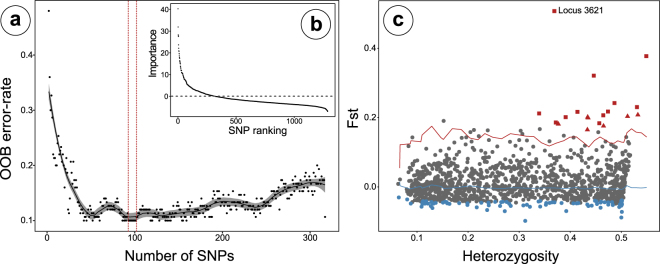
Detecting discriminatory loci using random forest and signals of selection. (**A**) Plot showing the results of the backwards purging approach, with the number of SNPs per subset plotted against the out-of-bag (OOB) error rate for each subset. The black line shows the smoothed estimates with 95% confidence-intervals (grey area). The two red dotted lines show the range of subsets (93–101 SNPs) with the lowest OOB error rate. (**B**) The inset shows the initial distribution of scaled importance values for each SNP before the backwards purging. The grey dotted line shows the importance threshold for the subset of SNPs used for backwards purging. (**C**) F_ST_ outlier analysis results showing individual SNP loci and 5% (blue line) and 95% (red line) confidence intervals. Outlier loci potentially under positive selection are in plotted in red and those potentially under balancing selection in blue. Squares mark F_ST_ outlier loci that were also detected as highly discriminatory using random forest and triangles those that are not shared. The significant outlier detected using BayeScan is labelled with ‘Locus 3621’.

**Figure 3:**
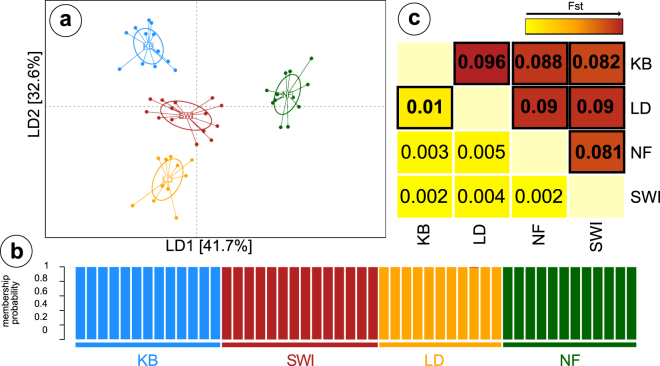
Population structure and population assignment in *L. salmonis* using discriminatory random forest loci. (**A**) DAPC plot of the first and second linear discriminant axis based on 98 highly discriminatory SNPs, explaining a total of 74.3% of the total variation. (**B**) Membership probability plot showing the population assignment probability for each individual. Each individual was correctly assigned to its sampling site. (**C**) Heatmap showing pairwise Fst between sampling sites based on the full SNP dataset (below diagonal) and based on the highly discriminatory SNP subset (above diagonal). Significant Fst values (inside each square) with P < 0.05 are highlighted in bold.

In order to detect population structuring among populations we utilised a random forest machine learning approach. We detected a subset of 93–101 SNPs that minimised the out-of-bag error rate to 0.1 (compared to 0.76 for the full dataset) and maximised the discriminatory power among populations (Fig. 2A). From this subset, we selected 98 SNPs for further downstream analyses. To assess the power of this subset of 98 highly discriminatory loci to detect significant population structure we performed the same population genetic analysis as was conducted on the full dataset. A PCA performed with the random forest selected subset showed a stronger separation between populations with a weak overlap of 95% confidence-intervals between LD and SWI (Figure S2, Supplementary data). However, the DAPC clearly separated all populations and the population assignment probability recovered was 1, meaning all individuals were correctly assigned to their respective population (Fig. 3). The variance explained among populations increased to 9.8% (from 1.8% with the full dataset) in the AMOVA (F_sc_ = 0.098, p < 0.0001). All pairwise comparisons showed highly significant Fst-values (range = 0.081–0.096), confirming the significant discriminatory power of the random forest detected SNP subset.

### Population discriminating SNPs and selection

One factor that might explain the strong discrimination of sea louse populations using the subset of random forest-selected SNPs would be divergent selection pressures, such as adaptation to different drug treatments, or local adaptation to natural environmental factors. Therefore, we performed two different tests for selecting SNPs under significant positive selection. An FDist approach implemented in Lositan detected 19 SNPs under strong positive selection (FDR < 0.1) with an average F_st_ of 0.233 ± 0.083 between populations (Fig. 2 C, Table 3). Eleven out of all 19 SNPs under positive selection are located on previously defined linkage groups 1 and 14, seven and four respectively^13^. The remaining SNPs are either located on linkage groups 4, 6 and 7 (two, two and one respectively) or could not be assigned to a linkage group. We further detected 46 SNPs under balancing selection (FDR < 0.1; p < 0.02).

**Table 3:** Outlier SNPs identified using the different approaches (Lositan, BayeScan and Random Forest) and annotation.

Locus ID	Contig_position	LG	Fst (Lositan)	Lositan	BayeScan	RF	Annotation
38173	lsalatl2s740_42780	4	0.320968	Yes	Putative	Yes	–
3621	lsalatl2s1185_140991	1	0.50726	Yes	Yes	Yes	–
40396	lsalatl2s80_965936	1	0.20663	Yes	No	Yes	PSA2
41679	lsalatl2s85_1109389	4	0.181416	Yes	No	No	—
42860	lsalatl2s907_144760	—	0.203467	Yes	No	No	—
4355	lsalatl2s122_618061	7	0.207882	Yes	No	No	—
6832	lsalatl2s139_1380660	1	0.199199	Yes	No	No	—
8287	lsalatl2s14_555303	1	0.377272	Yes	Putative	Yes	unchar.
8241	lsalatl2s14_1020918	1	0.217428	Yes	No	Yes	unchar.
9674	lsalatl2s163_163880	14	0.201546	Yes	No	No	—
15099	lsalatl2s228_333839	1	0.241913	Yes	No	Yes	—
1623	lsalatl2s10843_736	—	0.212014	Yes	No	Yes	—
21928	lsalatl2s3387_1782	—	0.185878	Yes	No	Yes	—
25024	lsalatl2s39_920686	6	0.216516	Yes	No	Yes	—
26383	lsalatl2s429_103294	14	0.175808	Yes	No	No	unchar.
29942	lsalatl2s514_325267	14	0.164932	Yes	No	No	—
30716	lsalatl2s535_184954	14	0.230334	Yes	No	Yes	—
2652	lsalatl2s1135_117353	1	0.183753	Yes	No	Yes	—
30805	lsalatl2s538_341294	6	0.20138	Yes	No	Yes	unchar.

Legend: RF stands for random forest, meaning SNPs that have been detected using the random forest approach. ‘Unchar.’ describes annotated genes that have not been characterized.

An analysis in BayeScan detected only one SNP (FDR < 0.01) under significant positive selection and two more putatively under selection (FDR < 0.05). All three of these SNPs were also detected to be under selection by Lositan and the significant one was also the top outlier in Lositan and located on linkage group 1 (F_st_ = 0.507). The other two putative SNPs in BayeScan were also highly significant in Lositan (p < 0.001) and were located on linkage group 1 and 4.

To detect how selection influences the genetic discrimination of populations we identified the amount of overlap between the 98 SNPs detected by random forest and all Lositan SNPs under significant positive selection. 63.2% of loci (12 out of 19 loci) detected to be under positive selection using Lositan were also identified being highly discriminatory between populations using random forest. Locus 3621, which was also identified using BayeScan, had the highest importance in the random forest analysis, suggesting that strong local adaptation and selection distinguishes sea louse populations.

### Annotation of outlier SNPs

In order to identify specific genes potentially involved in local adaptation and under positive selection in sea lice, we explored these regions in the annotated *L. salmonis* genome. Five of the 19 SNPs were in regions containing annotated genes within 10 kb, but only one of the annotated genes has been characterized. Two of the contigs with annotated genes were on linkage group 01, two on linkage group 14 and one on linkage group 6. Contig LSalAtl2s80 (linkage group 01) contained the characterized gene *PSA2*, which codes for the proteasome subunit alpha type protein.

### Validating the discriminatory power of random forest

The random forest classification algorithm identified a subset of 357 SNPs that minimised the out-of-bag error rate (0.324). 43 out of those 357 SNPs (12%) were labelled as ‘diagnostic’ SNPs that were included in the SNP array (419 diagnostic SNPs in total) to potentially distinguish populations across the Atlantic^13^. The assignment success to the geographic region of origin increased on average by 1.9% from 94.4% to 96.6% based on only this small subset of SNPs (see Table 4 for population specific values). In contrast to the IIb-RAD dataset the assignment success varied strongly across populations, ranging between 90.53–98.96% for the full SNP dataset and 91.58 to 100% for the random forest selected subset. The plots of linear discriminants from the DAPC analysis show a similar pattern, with the strongest separation of the Shetland and Irish populations from the remaining populations (Figure S4).

**Table 4:** Overview of sample origins and population assignment success for the full SNP dataset and the random forest (RF) subset for the Besnier *et al*. (2014) dataset^13^.

Region	Populations	Assignment % (Full)	Assignment % (RF)
Canada	C857	93.75	94.79
	C858		
Ireland	I852	97.92	97.92
	I853		
Faroe	F850	94.74	97.90
	F851		
Shetland	S855	98.96	100.00
	S856		
Southern Norway	N813	90.53	91.58
	N854		
Northern Norway	N837	90.63	95.83
	N849		

Even though the variation in population assignment success was greater for the larger dataset from Besnier *et al*. (2014), the random forest approach improved the assignment success for each population, supporting the applicability of this approach to different datasets.

## Discussion

In this study, we used a IIb-RAD sequencing approach coupled with advanced and sensitive population genetic analyses to genetically ‘fingerprint’ *L. salmonis* populations in the North-East Atlantic and to detect signatures of selection. We were able to achieve this using a relatively small (n = 50) number of individuals genotyped across only a limited portion of the genome (c.2.3Mbp = 0.34%). An important set of discriminatory loci was identified against a background of high genetic connectivity via a random forest machine-learning algorithm and these can be exploited to distinguish between nearby sea louse populations. A high degree of overlap between loci under positive selection using genome-scan approaches and loci with high discriminatory power from random forest analysis was also observed.

Sea lice are known to disperse rapidly among aquaculture sites as part of the larval zooplankton as well as via the movements of migratory (*Salmo salar)* or resident (*Salmo trutta*) anadromous salmonids^1^. Previous population genetic studies were consistent with such high levels of dispersal^8,10,12^, finding no significant genetic differentiation in our study region when utilizing a set of neutral microsatellite loci. Inclusion of putatively non-neutral loci can improve population discrimination across the Atlantic (e.g^10^.). However, the same studies could not distinguish populations on a small geographic scale as our data and approach suggest is possible.

More recent genome wide analysis of SNP variation in *L. salmonis* has to date been consistent with the lack of genetic structure that was found using classic markers such as microsatellite loci^13^. As with our dataset, correlation with geographic distances is not a feature of the genetic variation observed even with such genome-wide information. We found global F_ST_-values based on all loci to be significant but low (0.018), in agreement with patterns that have been found previously^13^. The use of anti-parasitic drugs has been shown to be a strong selective pressure in sea lice and several genomic regions under selection have been linked to drug treatment^13^. In particular linkage groups 1 and 5 in the study showed evidence of selective sweeps, with the same region on linkage group 5 being implicated in drug resistance in a QTL analysis^13^. Other linkage groups, such as 14 also showed signal of positive selection in that study^13^. Our study similarly found that 11 out of the 19 outlier loci we identified also lay in linkage groups 1 & 14, which represents an important independent validation. Spatio-temporal variation in treatment regimes, such as rotations of different drugs or the alternative use of warm-water or freshwater treatments^28^, may drive the heterogeneity observed in our and previous studies. This is partly as a result of cost, perceived efficacy, as well as different regulatory conditions in the countries concerned. Even though spatio-temporal variation in drug resistance is likely to be the strongest driver of differential selection among populations, local environmental conditions can constitute further selective pressures driving allele frequency differences among populations. Local environmental variables such as temperature (e.g^29^.) and salinity (e.g^30^.), for example, can have profound effects on sea louse survival and development. Furthermore, a combination of drug treatment and increased host density is shown to influence the evolution of reproductive and life history traits^31^, as well as virulence in sea lice^32^ among different populations. However, such local adaptation is most likely linked to subtle allele frequency differences, compared to strong selective sweeps caused by drug treatments, as the selective pressure is comparably low. The combination of a few outlier loci under strong positive selection and a wide range of loci showing subtle allele frequency differences fits the expected pattern. Independent of the cause for allele frequency differences among populations, we show that a random forest machine learning approach can be used to cost-effectively distinguish even near-by sea louse populations, even with a low number of samples and genotyping density.

The use of (historical) samples from the same site at different time points, differing in treatment regimes, could be used to disentangle the effects of drug regime and local adaption on allele frequency differences among populations and signatures of selection. Genome-scale population genetic profiling, alongside robust phenotyping, may also eventually reveal the genetic architecture underlying drug resistance and local adaptation. Here we have identified signals of selection across the genome, including markers closely associated with functional genes (e.g. *PSA2)*. The association of genomic response to selection, natural environmental conditions, and drug treatment profiles will be important considerations for future work.

Tools to enable parasite traceability and molecular epidemiology are an important requirement for rational sea louse control. Hydrographic modelling has been successfully deployed to understand short-range dispersal *L. salmonis* between farms and have been used to evaluate optimal treatment strategies^4,33^. Such model predictions can be biologically ‘truthed’ using planktonic trawls and strategically placed ‘sentinel’ fish that can infer the geographic scales of dispersal, as it has been done in one of the study areas, Kilkieran Bay (KB)^34^. However, biological (or genetic) confirmation of larger scale dispersal models (i.e. between lochs (=fjord) and loch systems) within and across regions is also required to assess long-range re-infestation risks for aquaculture sites. Such a strategy is of particular relevance in the light of an increasing control focus on loch-wide fallowing practices^35^. Furthermore, integration of genetic connectivity data with hydrographic larval dispersal models – so called ‘seascape genetics’ (e.g^36^.) - is likely to be more fruitful in defining any spatial-genetic correlations than crude map distances and represents an interesting further avenue for study. In this context, our data show that it may be possible to genetically ‘fingerprint’ louse populations in nearby regions to understand connectivity between them and provide a valuable tool for disease surveillance.

## Electronic supplementary material

Supplementary Dataset 1
