# Supplementary material for: Genetic fingerprinting of salmon louse (Lepeophtheirus salmonis) populations in the North-East Atlantic using a random forest classification approach
Source: Sci Rep. 2018 Jan 19;8:1203. doi: 10.1038/s41598-018-19323-z (PMC5775277; doi:10.1038/s41598-018-19323-z)
Supplement: Supplementary file 1 — Supplementary Dataset 1 [file 41598_2018_19323_MOESM1_ESM.doc]

**Supplementary material**

**Genetic fingerprinting of salmon louse (*Lepeophtheirus salmonis*) populations in the North-East Atlantic using a random forest classification approach.**

Jacobs A^1^, De Noia M^1^, Praebel K^2^, Kanstad-Hanssen Ø^3^, Paterno, M^4^, Jackson D^5^, McGinnity P^6^, Sturm A^7^, Elmer KR^1^, Llewellyn MS^1†^

Arne Jacobs: a.jacobs.1@research.gla.ac.uk

Michele De Noia: michele_dn@hotmail.it

Kim Praebel: kim.praebel@uit.no

Øyvind Kanstad-Hanssen: o-khan@online.no

Marta Paterno: martathaipat@gmail.com

Dave Jackson: Dave.Jackson@Marine.ie

Philip McGinnity: p.mcginnity@ucc.ie

Armin Sturm: armin.sturm@stir.ac.uk

Kathryn R. Elmer: kathryn.elmer@glasgow.ac.uk

Martin S. Llewellyn: martin.llewellyn@glasgow.ac.uk

† Corresponding author

Running head: Genetic fingerprinting of *L. salmonis* populations

**Figure S1:** Violin plots of Tajima’s D for each sea louse population.

**Figure S2:** Principal component analysis for the full SNP dataset and random forest candidate SNPs.

**Figure S3:** Distribution of scaled importance values for ranked SNPs for the Besnier et al. (2014) dataset.

**Figure S4:** Plots of linear discriminants 1 to 5 for the Besnier et al. (2014) dataset based on the random forest selected SNP subset.
